# Haemoglobin analysis on whole blood by reflectance photometry

**DOI:** 10.1155/S1463924685000438

**Published:** 1985

**Authors:** John A. Lott, Elias Khabbaza

**Affiliations:** Department of Pathology The Ohio State University Medical Center Doan Hall N-329 Columbus Ohio 43210 USA


Journal of Automatic Chemistry/*Journal of Clinical Laboratory Automation*, Vol. 7, No. 4 (October–December 1985), pp. 197–200

### Haemoglobin analysis on whole blood by reflectance photometry

**John A. Lott and Elias Khabbaza**

*Department of Pathology, The Ohio State University Medical Center, Doan Hall N-329, Columbus, Ohio 43210, USA*

### Introduction

New automated analytical methods in the clinical laboratory continue to appear, attesting to the development and maturation of the field. The progression has been from continuous flow analysers, to discrete analysers, and currently to 'reagentless' analysers (i.e. those using dry reagent-impregnated strips or pads) [1 and 2]. Stability under dry conditions, insensitivity to storage temperature fluctuations and a small, compact size are advantages of the dry reagent-impregnated strips or pads.

The purpose of this study was to evaluate the Seralyzer (Ames Division of Miles Laboratories, Elkhart, Indiana, USA) reflectance photometer and reagent strip system for the analysis of haemoglobin in whole blood. Three laboratories collaborated in this study, and each used the Coulter-S (Coulter Electronics, Inc., Hialeah, Florida, USA). The study was designed to test the comparability of the Seralyzer and Coulter-S haemoglobin methods on fresh patient specimens. Goals of the study included determining if there was between-method bias, estimating between-day precision, and testing for possible interferences from lipaemia, bilirubin, and carboxyhaemoglobin. Other objectives were to identify any critical steps in the procedure, limitations, and the applicability of the method for haemoglobin analysis in clinical laboratories.

### Materials and methods

### *Test strips*

The test strips consist of an absorbent reagent pad attached to a firm plastic support. The inert pads are impregnated with reagent, and each 100 g of impregnating reagent contains 50 g potassium ferricyanide, 19 g potassium dihydrogen phosphate, 19 g potassium monohydrogen phosphate and 12 g saponin.

The reaction used is:

$$\text{Haemoglobin} \xrightarrow{\text{ferricyanide}} \text{methemoglobin.}$$

Two lots of test strips were used at each of the three evaluation sites. Calibration was performed with solutions of D & C Red No. 33 dye (Ames) at 400 mg/l and 900 mg/l as described in the Seralyzer instrument manual [3]. The instrument was set at 7.2 and and 16.6 g/dl haemoglobin, respectively, with the dye solutions. The Coulter-S counters were calibrated for haemoglobin analysis using the well-known hemiglobincyanide spectrophotometric procedure recommended by the National Committee for Clinical Laboratory Standards (NCCLS) [4].

### *Instrument*

The reflectance of the strips was measured at 535 nm in the Seralyzer. The percentage reflectance of the pads was converted to concentration of haemoglobin with the simplified Kubelka-Munk [5] equation by the microprocessor in the instrument: $K/S = (1\text{-}R)^2/2R$, where $K$ is the molar absorptivity, $S$ is the scattering coefficient, and $R$ is the reflectance. $K/S$ is proportional to concentration. The optics and method of data reduction have been described in more detail elsewhere [1].

### *Method*

Well-mixed whole blood anticoagulated with 0.07 ml 150 g/l $K_3EDTA$ per 7.0 ml blood was used for analysis. Utilizing capillary action, a 10 µl glass capillary micropipette was filled with either whole blood or a control. After the outside of the capillary was wiped dry, the filled capillary was dropped into a 5 ml test-tube containing 800 µl of distilled water providing an 81-fold dilution of the whole blood. The tube was stoppered and mixed by agitation along the length of the capillary until a uniformly coloured solution was obtained.

After placing a strip on the strip carriage, 30 µl of the specimen was pipetted onto the centre of the pad. The start button was pushed, and the strip carriage was inserted into the Seralyzer. After approximately 60 s, the results were available on the digital display.

### *Comparison studies*

Fresh whole blood specimens from patients were reanalysed in duplicate on the Coulter-S and Seralyzer at all three locations. At the third site, the specimens were also analysed in duplicate on an IL 282 CO-Oximeter (Instrumentation Laboratories, Lexington, Massachusetts, USA).

### *Quality control*

Streck Hematology Controls I, II and III (Streck Laboratories, Omaha, Nebraska, USA) were used at all three sites. The approximate haemoglobin concentrations of the controls were 8 g/dl, 15 g/dl, and 22 g/dl, and the same lots of controls were used at all three sites. The controls were analysed in triplicate on each of the 20 days of the study at all three sites. The Streck controls' haemoglobin concentrations had been established with the NCCLS method [4].

*Table 1. Seralyzer versus Coulter-S: comparative data using fresh whole blood specimens.*

Site A

| Haemoglobin (g/dl) | *N* | Seralyzer mean | Coulter-S mean | Mean difference |
|---|---|---|---|---|
| <8·1 | 16 | 7·11 | 6·97 | +0·14 |
| 8·1–12 | 20 | 10·69 | 10·70 | −0·01 |
| 12·1–14 | 12 | 13·34 | 13·25 | +0·09 |
| 14·1–16 | 9 | 15·02 | 15·00 | +0·02 |
| 16·1–18 | 17 | 17·38 | 16·94 | +0·44 |
| 18·1–20 | 3 | 20·25 | 19·38 | +0·87 |
| Above 20 | 19 | 22·63 | 22·06 | +0·57 |

Site B

| Haemoglobin (g/dl) | *N* | Seralyzer mean | Coulter-S mean | Mean difference |
|---|---|---|---|---|
| <8·1 | 9 | 7·56 | 7·32 | +0·23 |
| 8·1–12 | 31 | 10·52 | 10·46 | +0·06 |
| 12·1–14 | 25 | 13·09 | 12·94 | +0·15 |
| 14·1–16 | 16 | 15·04 | 14·91 | +0·13 |
| 16·1–18 | 12 | 17·09 | 16·94 | +0·15 |
| 18·1–20 | 4 | 18·19 | 18·34 | −0·15 |

Site C

| Haemoglobin (g/dl) | *N* | Seralyzer mean | Coulter-S mean | IL 282 CO-Oximeter mean | Mean difference, Seralyzer from Coulter-S* |
|---|---|---|---|---|---|
| <8·1 | 8 | 7·28 | 7·32 | 7·21 | −0·04 |
| 8·1–12 | 42 | 10·12 | 10·20 | 10·14 | −0·08 |
| 12·1–14 | 21 | 12·98 | 13·02 | 12·97 | −0·04 |
| 14·1–16 | 15 | 14·76 | 14·86 | 14·70 | −0·10 |
| 16·1–18 | 16 | 16·64 | 16·71 | 16·57 | −0·07 |
| 18·1–20 | 3 | 19·12 | 18·57 | 18·72 | +0·55 |

* Mean of Seralyzer results (column 3) minus mean of Coulter-S results (column 4).

*Table 2. Correlation statistics on patient fresh blood analyses.*

| Site | Seralyzer (Y) *N* | Slope | Coulter-S (X) *N* | Intercept | Standard error | R |
|---|---|---|---|---|---|---|
| A | 96 | 1·04 | 96 | −0·31 | 0·54 | 0·99 |
| B | 97 | 0·99 | 97 | 0·21 | 0·39 | 0·99 |
| C | 105 | 1·01 | 105 | −0·14 | 0·44 | 0·99 |
| C | 105 | 1·00 | 105* | 0·01 | 0·53 | 0·98 |

* CO-Oximeter results.

### *Interference studies*

Lipaemic specimens, or those with total bilirubin concentrations between 4 and 32 mg/dl, were investigated for possible interferences with both the seralyzer and Coulter-S methods. For the lipaemic and icteric specimens, the cells were washed twice with a solution containing 154 mmol/l sodium chloride and 1.5 g/l $K_3EDTA$, and the volume was restored to the original volume with the same solution. The erythrocyte count of the specimens was used to determine whether any cells had been lost during washing. For almost all of the specimens, the count before and after washing agreed within 1 to 2%. Where these values differed by more than 1%, the haemoglobin concentration determined after the cell wash was multiplied by the factor: before wash count divided by after wash count. This was done to correct for minor cell loss in washing and to permit the accurate determination of the bilirubin and lipaemia interferences.

The possibility of interference from carbon monoxide was also investigated. Since interference by carboxyhaemoglobin in total haemoglobin assays occurs owing to the slow conversion of carboxyhaemoglobin to methemoglobin [6], the rate of conversion was investigated. After diluting 20 μl whole blood with 1.6 ml of distilled water, the sample was divided in half, and carbon monoxide was

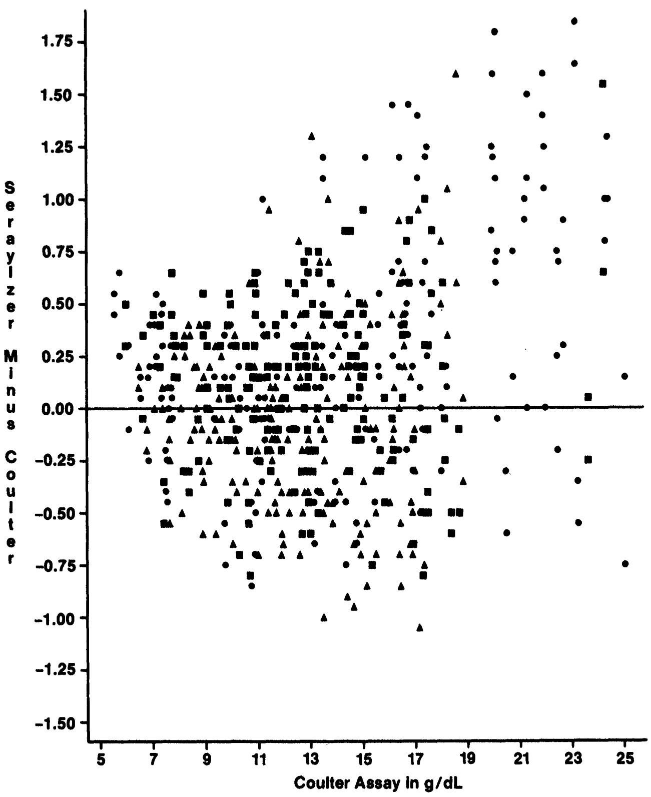

*Figure 1. Data on 298 patients analysed for haemoglobin by the Seralyzer and Coulter-S for site A (●), site B (■), and site C (▲).*

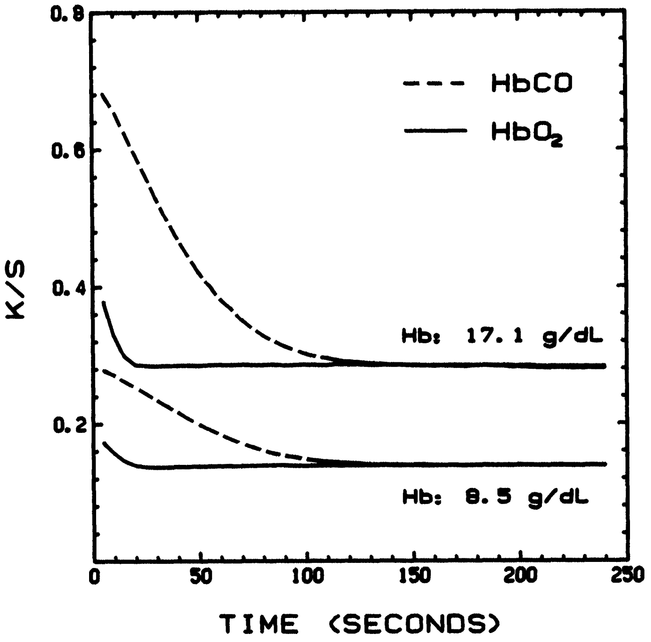

*Figure 2. Time profile of conversions of oxyhaemoglobin (solid line) or carboxyhaemoglobin (dashed line) to methemoglobin as determined by reflectance photometry and the K/S equation. The K/S value is proportional to concentration. Upper curve, haemoglobin of 17.1 g/dl; lower curve, haemoglobin of 8.5 g/dl.*

bubbled through one aliquot for 5 min. The two samples were analysed for haemoglobin on the Seralyzer, and the reflectance of the strip at 535 nm was followed over time.

### Results and discussion

### *Instrument*

The Seralyzer is easy to use and simple to calibrate. The approximate 1 min test time makes it suitable for smaller laboratories where only haemoglobin is measured.

### *Method*

The method was easy to perform; however, pipetting of the haemolysed and diluted blood onto the strips is a critical step. To obtain reproducible results, the specimen must be pipetted onto the middle of the strip. The reagent pad on the strip must not be touched by the pipette, as this may wash out the reagents or produce a pimple on the pad, both of which spoil the test. The strip must be inserted within 5 s of pushing the start button. Some training and practice is necessary for any new user when operating the instrument. Replicate results done at the same time should not differ by more than about 0.3 g/dl. A person with some familiarity with basic laboratory techniques should be able to perform the tests after reading the instruction manual [3], being shown how to perform the test, and then practicing the calibration and analysis technique for approximately 1 h.

*Table 3. Seralyzer haemoglobin precision: combined data from all three sites.*

| Control number | I | II | III |
|---|---|---|---|
| Number of days | 20 | 20 | 20 |
| Number of analyses | 183 | 183 | 117 |
| Mean | 8·2 | 14·5 | 21·1 |
| Within-run data | | | |
| SD | 0·18 | 0·31 | 0·52 |
| CV (%) | 2·2 | 2·1 | 2·5 |
| Between-run data | | | |
| SD | 0·16 | 0·28 | 0·45 |
| CV (%) | 2·0 | 1·9 | 2·1 |

### *Comparative data on patient specimens*

Comparative data obtained on fresh whole blood specimens at the three sites are shown in table 1, and the correlation statistics are detailed in table 2. The reference instruments (Coulter-S) were calibrated with different sources of haemoglobin at the three sites. Given the excellent agreement between the Seralyzer and the Coulter instruments at all sites, it is reasonable to conclude that the Seralyzer gives results that are a good estimate of the true haemoglobin concentrations. Using an IL CO-Oximeter at site C, precise results were confirmed since agreement with the Seralyzer was very good. Figure 1 shows the differences between the Seralyzer and Coulter-S haemoglobin determinations on 298 fresh patient specimens at the three sites. Below 19 g/dl hemoglobin no statistically significant bias existed between the Seralyzer or the Coulter-S.

The Seralyzer cannot be used for specimens with less than 5 g/dl haemoglobin. Above 19 g/dl hemoglobin, the

*Table 4. Haemoglobin interference studies.*

| | Coulter-S | | Seralyzer | |
|---|---|---|---|---|
| | Before wash (g/dl) | After wash (g/dl) | Before wash (g/dl) | After wash* (g/dl) |
| Specimens | | | | |
| *Icteric* ($N$ = 22) | | | | |
| Mean | 11·44 | 11·35 | 11·70 | 11·66 |
| SD | 1·85 | 1·87 | 1·86 | 1·95 |
| *Lipemic* ($N$ = 9) | | | | |
| Mean | 12·36 | 12·16 | 12·23 | 12·23 |
| SD | 3·34 | 3·27 | 3·35 | 3·24 |

*Note:* The sodium chloride-EDTA solution contained 154 mmol/L sodium chloride and 1.5 g/l $K_3EDTA$. See text for details.

* Corrected for cell loss during washing

Seralyzer showed a significant positive bias at sites A and C (table 1). Specimens with a haemoglobin above 19 g/dl should be diluted with 154 mmol/l sodium chloride prior to analysis on the Seralyzer.

*Precision*

No statistically significant between-lot variation of the strips was observed for the Seralyzer results on the Streck controls I, II and III, so the data from the three sites were merged. Also there was no bias by the t-test between the three sites for the control results (table 3). Controls I and II were analysed in triplicate on each of 20 days, and control III was analysed on each of about 10 days at each site. The within-run standard deviation (SD) was calculated each day; the within-run SDs in table 3 are the averages of the within-run SDs.

The within-day means were calculated for each control, and these data were used to calculate the between-run SDs. The use of the means is probably the reason why the between-day SDs are slightly smaller than the within-run SDs. The between-run average coefficient of variation (CV) of about 2% is well within the medically acceptable limits of 3% at 8 to 21 g/dl haemoglobin [7].

*Interference study*

The effects of bilirubin and lipaemia are summarized in table 4. The effect of bilirubin is very slight with both instruments. Specimens with 30 mg/dl, 31 mg/dl, and 32 mg/dl bilirubin showed at most a haemoglobin which was 0.3 g/dl higher with the Coulter-S before washing. The Seralyzer method was unaffected by bilirubin. The effect of lipaemia is negligible, and the difference seen before and after washing the cells was due to experimental error.

Carbon monoxide does not interfere in the determination of haemoglobin; however, carboxyhaemoglobin is converted to methemoglobin more slowly than oxyhaemoglobin (figure 2). The seralyzer does not display a result until a stable reflectance is obtained. In the presence of large amounts of carboxyhaemoglobin, in may take 2 to 3 min before a result is obtained.

### Conclusions

Using dry reagent strips, the Seralyzer provides medically acceptable results for whole blood haemoglobin. The usable analytical range of the instrument is 5 to 19 g/dl. Neither bilirubin, carbon monoxide, nor lipaemia interfere.

### Acknowledgements

The authors wish to thank Dan Wong of Ames for the carboxyhaemoglobin interference study and Larry W. Clark for technical assistance and for reviewing the manuscript. Budd Mott (Cushing Municipal Hospital, Cushing, Oklahoma, 74023, USA) provided the data from site B, and Dr Michael Shehan (Good Samaritan Hospital, Portland, Oregon, 97210, USA) provided the data from site C.

### References

1. Karmen, A. and Lent, R., *The Journal of Clinical Laboratory Automation*, **2** (1982), 284.
2. Bandi, Z. L., Fuller, J. B., Bee, D. E. and James, G. P., *Clinical Chemistry*, **27** (1981), 27.
3. *Instruction Manual, Ames Seralyzer* (Ames Company, Division of Miles Laboratories, Elkhart, Indiana, USA).
4. National Committee for Clinical Laboratory Standards, *Standard H7-T: Hemiglobincyanide Spectrophotometric Procedure for Hemoglobinometry Standardization* (NCCLS, Villanova, Pennsylvania, 1979).
5. Kortuem, G., *Reflectance Spectroscopy* (New York, Springer Verlag, 1969), p. 106.
6. Taylor, J. D. and Miller, J. D. M., *American Journal of Clinical Pathology*, **43** (1965), 265.
7. Barnett, R. N., *American Journal of Clinical Pathology*, **50** (1968), 671.

